# Duties of the Medical Profession

**Published:** 1897-09

**Authors:** 


### Duties of the Medical Profession.

Dr. Hamilton, *Journal American Medical Association*, speaking of the duty of the medical profession, says:

"There are several methods whereby medical men can influence their administrative and legislative servants. 1. The first and most important is by corporate action, resolutions, committees, etc., through medical societies. Strange as it may seem, this, by some physicians, is considered unprofessional and *ultra vires.* The very thing we should do, that precise method of making our influence felt which is most effective and most imperatively demanded is, wonderful to say, actually the thing at which we balk and stop. No matter what disgrace to medicine may be threatened, no matter how clear the professional duty that confronts us, there are to-day not a few of the so-called leaders—professional tories and aristocrats, they might be termed—who say, "Oh, yes! It's all a miserable shame, but *non possumus!* Our organization gives us no right or power to go into politics." Bah! This is the voice of laziness or cowardice; it is not the way of scientific advance or of the cure and prevention of disease. Medical societies have not only the right and power, they have the inobviable duty laid upon them, shirk it and deny it as they may, to make their influence felt in all public questions relating to the public health. The work of the British Medical Association and of its parliamentary and other committees in eradicating abuses and in instituting reforms, gives the lie to our pusillanimity and, by example, illustrates what may be done by men whose motives are pure and whose bravery puts their motive into action. Every American medical society from the smallest to the largest is under the most stringent obligations to make matters of the public health its most direct concern. Each one, and especially the American Medical Association and the Medical Congress, should pass resolutions pertaining to bad laws proposed or to good laws that should be proposed in reference to medical education, quarantine, health-protective measures, etc., and should see that copies of these resolutions are placed in the hands of every legislator and administrator whom they concern. Only thus shall we make our professional will and power known, and in becoming known it shall bring about reform and social education. Our organizations shall also appoint committees, and see that these committees do their work to bring to the consciences and consciousnesses of the public officials their duties as to the prevention and cure of disease.

"2. Not only by means of our organizations can we further our cause, but hardly less effective, if strenuously carried out, would be the work of individuals of the profession, should they try to influence their lawmakers and administrators in a private way. If these men are known to us personally, by all means protest and advise by word of mouth. Incalculable good could be done so. If the powers that be are not our acquaintances or patients, there remains the letter carrier to help us. And a powerful influence is the written letter! When a vicious piece of legislation is proposed, or when good measures are neglected, we should write to legislators and governors and mayors in condemnation of its commendation. Many a shameless bit of medical jobbery is smuggled into law simply because the men with power hear only the quacks' side and not a word is uttered by us who are the appointed guardians of the nation's health. The few quacks combine, hire a lobby, write a thousand letters, appoint committees, personally intercede and win the day; while we, who are 100,000, meet in a thousand societies, local, State and National, and talk about headaches, sprains and sore toes, but never a word about prevention or cure of the common professional and social miseries that afflict and murder millions. The profession by such silence is not only renegade to its humanitarian function, but it is renegade to its own self-interest. A man may not always be his brother's keeper, but a physician is always so, and when he denies or neglects the fact he is a shirker, and at heart and in fact an enemy of his own guild.

"While we acquiesce in silence, physicians who value their selfish aims and interests above those of the world and of their profession, form a compact and make a raid on the public treasuries for endowments and 'divvies' for the benefit of hospitals, dispensaries and medical colleges founded and carried on not for the public good but for the benefit and profit of the lobbyist.

All the time there is not one in a thousand of the epileptics of the land cared for, his life turned from a curse to a blessing by the one sure method which we all know. Is this medicine? Is it humanity? A hundred such examples of abuse and disgracefulness arise spontaneously in every reader's mind, and need no further listing. The antivivisectionists, the meat-sellers and milk-dealers who are only kept from sewing the seeds of disease by inspectors and legal control, go on with their infamous traffic because, forsooth, we do not care or because the stoppage of the misery is *ultra vires!* Can a more flagrant example of professional cowardice and stultification be cited? Shall it go on forever, while eternally postponing we sing the old woman's song:

'Consider, good cow, consider!'"

